# *“COVID gave him an opportunity to tighten the reins around my throat”*: perceptions of COVID-19 movement restrictions among survivors of intimate partner violence

**DOI:** 10.1186/s12889-023-15137-5

**Published:** 2023-01-30

**Authors:** Kathryn G. Wyckoff, Subasri Narasimhan, Kaylee Stephenson, Amy J. Zeidan, Randi N. Smith, Dabney P. Evans

**Affiliations:** 1 Department of Behavioral Social and Health Education Sciences, Rollins School of Public Health, Emory University, 1518 Clifton Rd, 30322 Atlanta, USA; 2 Hubert Department of Global Health, Rollins School of Public Health, Emory University, 1518 Clifton Rd, 30322 Atlanta, USA; 3 Emory School of Medicine, Department of Emergency Medicine, Atlanta, USA; 4 Emory School of Medicine, Department of Surgery, Atlanta, USA

**Keywords:** Intimate Partner violence, Domestic violence, COVID-19, Pandemic, Movement Restrictions

## Abstract

**Background:**

Intimate Partner Violence (IPV) poses a serious public health threat globally and within the United States. Preliminary evidence highlighted surges in IPV during the COVID-19 pandemic. The pandemic offers a unique context, with many states and countries enacting movement-restrictions (i.e., shelter-in-place orders) that exacerbated IPV. Although these movement restrictions and other infection control methods (i.e., isolation, quarantine orders) have proven successful in reducing the spread of COVID-19, their impacts on IPV have not been thoroughly investigated. Specifically, public health measures restricting movement reinforce and socially legitimize isolation and coercive control tactics enacted by perpetrators of abuse. The purpose of this study was to understand the impacts of COVID-19, including the impacts of movement restrictions (i.e., shelter in place orders, quarantine, isolation orders) on experiences of IPV from the perspective of survivors.

**Methods:**

In-depth interviews were conducted with ten survivors who presented at a large, public hospital or sought community IPV resources (i.e., domestic violence shelter, therapy services) in Atlanta, Georgia between March and December 2020. Thematic analysis was carried out to describe the impact of COVID-19 movement restrictions on IPV and help-seeking behaviors among survivors, in addition to identifying resources to improve IPV response during pandemics.

**Results:**

Through discussion of their experiences, survivors indicated how movement restrictions, social distancing measures, and the repercussions of the pandemic influenced their relationship challenges, including the occurrence of new or a higher frequency and/or severity of IPV episodes. Survivors cited relationship challenges that were amplified by either movement restrictions or consequences of COVID-19, including reinforced control tactics, and increased financial or life stressors resulting from the pandemic. COVID-19 movement restrictions catalyzed new relationships quickly and sparked new or intensified violence in existing relationships, revealing gaps in IPV support services.

**Conclusion:**

These findings suggest COVID-19 movement restrictions and social distancing measures amplify IPV and experiences of trauma due to new or exacerbated relationship challenges. Further, results highlight how partners cited COVID-19 movement restrictions to justify methods of coercive control. Public health professionals engaged in pandemic preparedness must give serious consideration to how social distancing measures may amplify trauma in those experiencing IPV.

## Introduction

A broad range of research examining COVID-19 and intimate partner violence (IPV) has emerged since the onset of the pandemic with several systematic reviews finding increases in IPV especially during lockdown and social distancing periods [1–3]. Despite the positive intention to mitigate the negative effects of COVID-19, movement restrictions (i.e., shelter-in-place orders, school closures, curfews) effectively trap survivors with their abusers by creating isolating environments and exacerbating coercive control tactics [4–6].

Anecdotal evidence and commentaries early in the pandemic rang the alarm bell about the “shadow pandemic.” [7]. Several studies highlighted existing disparities that might be exacerbated by the pandemic, such as economic instability, unsafe housing, neighborhood violence, and low social support [4–6, 8]. They also explored potential explanations for decreases in help-seeking behaviors or calls to crisis lines due to lack of safety in connecting with resources due to sheltering in place with perpetrators of IPV [4, 5, 9]. Diminished IPV resources and shelters with reduced capacity, overburdened health care systems and providers, reduced social support (i.e., family, friends), as well as limited or reduced capacity law enforcement means (i.e., protective orders) left survivors of IPV in a particularly vulnerable place [4, 5, 9]. Such factors necessitated research into the relationship between movement restrictions, help-seeking behaviors, and IPV during COVID-19. Preliminary research on this topic has proven these concerns to be warranted [9].

Research at the onset of the pandemic suggested ways that it was negatively affecting relationships [1]. Across the United States (US), cross-sectional research suggested increases in violence reports during perpetrator working hours [10], increases in calls to service organizations [11] and police [12] during lockdown. Others found an absence of increases in reported assaults despite increases in calls to service organizations [13], decreases in reported IPV cases [14] and increased odds of experiencing IPV among those with COVID-19 symptoms or diagnosis [15]. Of note, crime data analyzed from the Atlanta Police Department revealed increases in domestic crimes during 2020 compared to the previous two years, suggesting increases in domestic violence [16]. These findings are particularly pertinent, as the current study takes place in Atlanta, Georgia.

Several cross-sectional studies have underscored the need for research on IPV risk factors during COVID-19 and the effects of movement restrictions from the perspectives of survivors [17]. A cross-sectional study examining U.S. emergency room visits for mental health, overdose, and violence outcomes between 2018 and 2020 noted increases in visits pertaining to IPV during the lockdown period compared to previous years, indicating the severity of violence warranted breaking movement restrictions and navigating health risks of COVID-19 [18]. Research in Spain also found lockdown and economic stress factors significantly contributed to increases in IPV, suggesting lifted movement restrictions would not decrease IPV due to continued economic stress fueled by the pandemic [2].

Other studies have explored adverse mental health outcomes related to movement restrictions. A large cross-sectional study in the United Kingdom (UK) found higher levels of anxiety and depression during the initial COVID-19 lockdown period [19]. Another large cross-sectional UK study found significant relationships between experiences of previous physical and/or psychological abuse, pre-existing mental health issues, decreased social support, and low socioeconomic status and the onset of depressive symptoms during the COVID-19 lockdown period between March and April 2020 [20].

Evidence from qualitative research with female IPV survivors in three countries suggest COVID-19 increased individual stressors, such as financial stress or unemployment [8, 21–23] mental health complications, [8, 21, 22], household work and caregiving burdens [21–23], as well as increased severity and incidence of IPV associated with increased alcohol consumption [22], control tactics (i.e., partner isolation, control of movements, monitoring) [21–23], and confinement within the home [8, 21–23]. Additionally, data from survivors interacting with shelters and IPV service agencies suggest a lack of support from shelters during the pandemic, in addition to exacerbated feelings of isolation stemming from a combination of strict shelter rules and stay-at-home orders that mirror control or isolation tactics enacted by abusive partners [8].

A plethora of research examining IPV during the COVID-19 pandemic has been published in the US since 2020 [24]. A systematic review characterized studies into the subcategories of: (1) victimization, (2) perpetration, (3) victimization and perpetration, and (4) provider perspectives [24]. Of the 53 articles included in this systematic review, only four studies included qualitative in-depth interviews with survivors of IPV examining victimization specifically [21, 24–26]. Notably, survivors recruited for these studies were from the Pacific Northwest [25], Texas [26], as well as immigrants residing in Massachusetts, New Jersey, Texas, Illinois, Maryland, Virginia and Washington DC [21]. Therefore, the landscape of research examining IPV during the COVID-19 pandemic is still missing largely the perspectives of survivors, especially in the Southeast region of the US.

As the COVID-19 pandemic continues, its long-term effects on the experiences of IPV survivors are still largely unknown, including gaps in understanding the overarching impacts of quarantining and movement restrictions on survivor experiences. The perspectives of IPV survivors who sought healthcare or resources pertaining to their relationship during COVID-19 are largely absent from current information and their perspectives are necessary to provide insight into the pandemic’s impact on IPV and help-seeking behaviors [27, 28]. The current study aims to address the gap of qualitative in-depth interviews with survivors of IPV during the COVID-19 pandemic. The purpose of this study was to understand the impacts of COVID-19, including the impacts of movement restrictions (i.e., shelter in place orders, quarantine, isolation orders), on experiences of IPV from the perspective of survivors. Exploring this unique perspective provides necessary context to existing evidence.

## Methods

### Design

The study utilized a qualitative design embedded within a larger mixed-methods study. As a relatively novel area of research, the study design maximized the investigation of the impacts of COVID-19-related movement restrictions on IPV and experiences of trauma, health-seeking, and community resource-seeking behaviors from the perspectives of IPV survivors, the group directly affected. As IPV is a sensitive topic, in-depth qualitative interviews were selected, as they prove useful in building rapport and eliciting perceptions and experiences from survivors.

### Study site

Of the 80,921,000 men and women estimated nationally to have experienced IPV in their lifetime [27], 14.5 million estimated lifetime survivors reside in the state of Georgia where this study takes place [28]. Prior research underscores the high prevalence of IPV in Georgia [28], in addition to its rankings as one of the states with higher rates at which women are killed by men [29]. On March 14, 2020, Georgia Governor Brian Kemp issued Executive Order No. 03.14.20.01 [30], which declared a Public Health State of Emergency in Georgia and called for the enactment of social distancing measures. On behalf of the City of Atlanta, Mayor Keisha Lance Bottoms issued Executive Order No. 2020-21 [31] on March 23, 2020, thereby enacting a citywide shelter in place order. The following week, Governor Kemp issued Executive Order 04.02.20.01 [32] on April 2, 2020, enacting a statewide shelter in place order. In compliance with these orders, residents were instructed to stay in their homes, leaving only to carry out essential business, during which they were advised to practice social distancing measures. Although these movement restrictions and other infection control methods (i.e., isolation, quarantine orders) were important measures for reducing the spread of COVID-19, their impacts on IPV were unknown at the time, with many speculating that such restrictions could place individuals at heightened risk [17]. Cumulative increases in 2020 domestic violence (DV) crimes in the city of Atlanta compared to the previous two years highlight surges of IPV during the pandemic [16]. As two counties within the Atlanta area contain the highest prevalence of COVID-19 cases, hospitalizations, and total deaths for the state [33], Atlanta, Georgia provides an ideal context in which to examine the effects of the COVID-19 pandemic on IPV from the perspective of survivors for this study.

### Instrument

Two original in-depth interview (IDI) guides were created, one for IPV survivors recruited using electronic medical records from a large public hospital, and one for IPV survivors recruited from the community in Metropolitan Atlanta, Georgia. Both guides included questions designed to explore the following topics: knowledge and perceptions of COVID-19 and movement restrictions, perceptions and experiences of IPV during the COVID-19 pandemic perceived effects of COVID-19 movement restrictions on experiences of IPV 4), if relevant, perceived changes in IPV experiences from before compared to during the COVID-19 pandemic, facilitators for experiencing IPV during the COVID-19 pandemic, perceptions of facilitators and barriers to seeking IPV resources during the pandemic, and perceptions of resources or supports that could improve IPV response during pandemics. Each guide was divided into seven sections. Section one included quantitative questions to collect survivor demographic information. Section two included a mixture of quantitative and qualitative questions concerning knowledge and perceptions of COVID-19 and movement restrictions across Atlanta and the state of Georgia, as a whole. Section three consisted of qualitative questions about survivors’ lives and challenges prior to and during the COVID-19 pandemic. Section four contained qualitative questions concerning survivors’ current or most recent relationships, their relationship challenges prior to and during the pandemic, as well as experiences of IPV before and during the pandemic. Section five differed slightly for survivors recruited from the hospital versus survivors recruited from the community, in that survivors recruited from electronic medical records were asked questions concerning their visit to the hospital following IPV; survivors recruited from the community were asked questions about the social services (i.e., domestic violence shelter, therapy services) they sought for IPV during the pandemic. Section six included quantitative and qualitative questions about survivors’ current and previous experiences with IPV, knowledge of available resources, and remaining resource needs. Lastly, section seven included wrap-up questions to debrief survivors, share IPV resources, and close the interview.

The lead interviewer held a doctoral degree in public health and had extensive qualitative research experience including with survivors of gender-based violence. The secondary interviewer was a Master of Public Health graduate student at the time of data collection. Her training included advanced courses designed to develop mastery of a variety of practical techniques and theoretical approaches to qualitative data collection and analysis.

The lead interviewer pilot tested both IDIs with members of the research team and feedback from practice interviews were incorporated into the final guides, which also included probing techniques to extract additional information from participants. Once pilot testing was complete and the first set of patient and community survivor interviews were conducted, the research team made iterative changes to the IDIs, including the addition of probes and inclusion of language stressing the importance of taking the interview in a private space away from family members or intimate partners.

### Participants

To be eligible for study participation, survivors had to have presented at a large, Atlanta-based public hospital between March 2020 and December 2020 or have sought IPV community resources (i.e., domestic violence shelter, psychosocial therapeutic services) during the same time frame. Additionally, during recruitment phone calls, survivors needed to screen positive for IPV, including physical violence, sexual violence, stalking and psychological aggression by a current or former intimate partner. For patient survivors, we used International Classifications of Disease (ICD)-9 and − 10 codes as part of the larger parent study where we developed a natural language processing algorithm to identify IPV survivors. Their process and analysis related to that portion of the study has been described elsewhere [34, 35]. Ultimately, we had an eligible sample of 172 patients, of which only 2.9% were successfully recruited after a nine-month recruitment period with three follow-ups per participant. To diversify the sample, the research team used social media advertisements and reached out to community providers of IPV resources to distribute study fliers among groups and listservs to recruit interested community members with IPV experiences during the COVID-19 pandemic. Due to the sensitive and challenging nature of recruiting people actively experiencing relationship violence, all interested and eligible participants who met the inclusion criteria were included in the final sample (n = 10).

Survivors were recruited using text messages sent to patient phone numbers obtained through the patient’s electronic medical record. To ensure survivor safety, initial text messages informed patients they may qualify to participate in an Emory University study. If there was no reply, the research team followed up with a text message every three days for three attempts. Following a reply expressing interest, a short phone call which described the study as a “COVID-19 and Personal Health” was scheduled to confirm patient identity and eligibility, explain the study’s purpose, schedule a date and time for a Zoom interview, and set up an identity passphrase for use during the interview to ensure safety. To ensure survivor safety, no voicemail messages were left if patients did not answer phone calls.

Survivors from the community were recruited for the study via a distribution of a study flier containing eligibility requirements, study information, and contact information for members of the study team with whom interested individuals could reach out to. Study fliers referring to the study as about “COVID-19 and relationships” were distributed via social media advertisements, listservs from Atlanta-based IPV organizations, and public spaces (i.e., public transit stations, grocery stores, shopping malls, parks). Once contacted, members of the study team set up a five-minute phone call to confirm eligibility, explain the study’s purpose, schedule a date and time for a Zoom interview, and set up an identity passphrase for use during the interview to ensure safety. As with other participants no voicemail messages were left if community members did not answer phone calls. All participants received confirmation texts and emails with the study’s informed consent and Zoom invite, in addition to an interview reminder 24 hours in advance. All participants were provided with links to a secure safety planning app, and received a password-protected IPV resource list. All participants were compensated with a $25 gift card following interview completion.

### Data collection

Data collection occurred between April 2021 through January 2022. Despite data collection occurring at a single time point, the study examined IPV survivors’ perceptions and experiences prior to and during the COVID-19 pandemic due to inclusion criteria of participants. Potential issues of recall bias were mitigated by incorporating time frames into the interview guide for participants to focus their responses.

Following pilot testing, two interviewers conducted ten in-depth interviews with IPV survivors, five with survivors recruited from the hospital and five with survivors who sought IPV resources in the community. In addition to the interviewer, another member of the research team was also present for each interview to take field notes. Interviews were conducted and recorded remotely via Zoom and lasted between 60 and 120 min. Two of the ten interviews were interrupted and rescheduled due to the presence of an abuser or a third party that compromised the privacy of survivors. To ensure privacy and safety were maintained, a safe phrase was established with survivors prior to each interview that they could use to end the interview at any time. Following each interview, verbatim transcripts were produced using HappyScribe [36], with quality checks conducted by a graduate research assistant. At the end of each interview, survivors were provided with links to a secure safety planning app and a password-protected IPV resource guide containing local resources.

### Data analysis

Data management and analysis were carried out using MAXQDA Analytics Pro 2022 [37]. Thematic analysis was selected as the analysis framework. Thematic analysis refers to “the method for identifying, analyzing, and reporting patterns (themes) within data.” [38] For this study, the phases of thematic analysis identified by Braun and Clarke were employed [38]. These phases include data familiarization, creating initial codes, searching for themes, reviewing themes, defining themes, and creating a final report [38]. In order to advance data familiarization, a thorough review of all transcripts was completed. Throughout the course of data orientation and subsequent analysis, the process of memoing, writing annotations or comments, was employed to keep an audit trail of analytical decisions, notes, methods employed, and to develop final themes.

Prior to the identification of codes, the graduate research assistant reoriented themselves to the overarching research goal to describe the impact of COVID-19-related movement restrictions on the experiences of trauma resulting from IPV, including health or help-seeking behaviors. During the code identification process, codes were developed deductively using domains from the IDI guides and IPV literature; inductive codes were developed as part of the data familiarization and preliminary memoing processes. Several initial codes came from breaking interview questions down into smaller pieces and some of the examples brainstormed during initial codebook discussions with the larger research team. Examples of deductive codes included “COVID-19 insights,” “COVID relationship challenges,” “IPV classification,” and “Negative help-seeking experiences.” This method aligns with Bazeley’s [39] approach to organizing code structures based on conceptual similarities, while also ensuring that each concept only appeared in the code structure one time. Inductive codes were also developed between the two interviewers based off recurring topics from interviews. Examples of inductive codes include “Financial control,” “First-time relationship violence,” and “Substance use.”

Following coding of the first transcript, additional inductive codes were added, and the final codebook underwent review by the larger research team. The finalized codebook was then used to recode the first transcript and subsequent nine transcripts. After coding was completed, a variety of methods were employed throughout primary data analysis and theme development. These methods include memoing, case summaries, reflections, matrices, as well as comparisons across data. Finally, descriptive statistics were run on quantitative data using Qualtrics and Excel.

### Ethical considerations

This study was approved by Emory University’s Institutional Review Board (Study ID 00000432). Informed consent forms were emailed or texted to participants based on their preferences in advance of interviews and read aloud to participants prior to the start of each interview. Verbal consent was obtained and documented by the research team for each participant prior to data collection. Survivors were also provided with access to a secure safety planning app and a password-protected IPV resource guide containing local resources (e.g., hotlines, DV shelters, general DV resources, temporary housing, health care, legal assistance) to minimize study harms, potential retraumatization, and ongoing IPV. The research team utilized guidance from the World Health Organization on conducting research on violence against women throughout the study [40].

## Results

Participants interviewed included 8 female-identifying survivors, 1 male-identifying survivor, and 1 non-binary survivor. The majority of survivors interviewed identified as Black or African American (n = 7) while others identified as Multiracial (n = 2) or White (n = 1). The mean age of survivors was 37 years of age. Half of survivors interviewed were self-reported as single, not in a relationship, at the time of their interview (n = 5). Four survivors had a private insurance plan during 2020 (n = 4). When asked about services sought, five survivors reported obtaining services at the large, Atlanta-based public hospital, two sought services at domestic violence shelters, and three sought therapy services following IPV during the pandemic. Seven of the survivors interviewed indicated awareness of Atlanta- or Georgia-specific COVID-19-related movement restrictions (Table 1).

**Table 1:** Survivor Demographic Information

Characteristics	Overall N = 10
Age in years, mean (SD)	37.4 (14.23)
**Gender, n (%)** Female Male Non-binary	8 (80) 1 (10) 1 (10)
**Race, n (%)** Black or African American Multiracial White	7 (70) 2 (20) 1 (10)
**Children, n (%)** Yes No	5 (50) 5 (40)
**Relationship Status at time of Interview, n (%)** Single Married In a Relationship Separated	5 (50) 2 (20) 2 (20) 1 (10)
**2020 Insurance Status, n (%)** Private Plan Medicaid Uninsured TRICARE	4 (40) 3 (30) 2 (20) 1 (10)
**Employment Status at time of interview, n (%)** Full-time Unemployed Part-time	5 (50) 3 (30) 2 (20)
**Primary services sought following IPV, n (%)** Hospital Therapy DV Shelter	5 (50) 3 (30) 2 (20)
**Knowledge of Atlanta- or Georgia-specific COVID-19 movement restrictions, n (%)** Yes No	7 (70) 3 (30)

We identified two themes describing the impact of COVID-19 movement restrictions and social distancing measures on IPV survivor experiences of trauma and help-seeking behaviors: COVID-19 paved the way for relationship challenges catalyzing violence, and COVID-19 movement restrictions catalyzed new relationships quickly and sparked new or intensified violence in existing relationships. Within each theme, subthemes provide further elaboration and explanation.

### COVID-19 paved the way for relationship challenges catalyzing violence

Each of the survivors cited relationship challenges that were amplified by either movement restrictions or the consequences of COVID-19. Dimensions of these recurring relationship challenges include increased substance use, reinforced tactics of control or abuse, as well as increased financial or life stressors resulting from the pandemic. Notably, survivors attributed these COVID-19 related relationship challenges to higher recurrences of arguments or fights, which often preceded episodes of IPV. Within this theme two subthemes were identified: COVID-19 movement restrictions and social distancing measures reinforced control and abuse tactics contributing to relationship challenges and IPV; and COVID-19 restrictions and impacts pertaining to financial and life stressors worsened IPV.

#### COVID-19 movement restrictions and social distancing measures reinforced control and abuse tactics contributing to relationship challenges and IPV

COVID-19 movement restrictions and social distancing measures bolstered perpetrator methods of control over survivors. Ensuing episodes of IPV stemmed from the challenges and arguments resulting from these reinforced control or abuse tactics. Among relevant cases, COVID-19 movement restrictions and social distancing measures either overtly or covertly augmented survivor experiences of IPV.

Perpetrators overtly mechanized COVID-19 movement restrictions to reinforce control tactics with one survivor noting that her partner was extremely paranoid about COVID-19 and claimed he did not want anyone coming to the house or the survivor leaving the house. She described him calling her from work every hour to make sure she was home and using COVID-19 to justify installing a tracker on her smart phone to ensure she was not leaving the house. When questioned about if the survivor thought these coercive control tactics were used out of fear of COVID or desire to control her, she indicated:

*“Control. COVID gave him an opportunity to tighten the reins, if you will, around my throat.” (39-year-old Multiracial female survivor)*

As a result, the intensified control measures reinforced by COVID-19 restrictions contributed to increased arguments when the survivor left the home against the perpetrator’s wishes, which were followed by episodes of physical IPV.

Conversely, survivors discussed instances in which COVID-19 movement restrictions and social distancing measures more subtly contributed to survivor experiences of IPV. One male survivor indicated his long-term partner had issues with jealousy. His partner had cheated on him previously and her feelings of jealousy were amplified during the pandemic:

*“She would do crazy stuff like delete all my contacts from my job…church female members and stuff like that. She would erase their phone numbers out my cell phone. My…daughter’s number, her mother’s number, my sister’s number…any female phone number that she found on my phone, she would delete it.” (65-year-old Black/African American male survivor)*

Other survivors discussed how, through a combination of COVID-19 movement restrictions and their partner’s actions, they were isolated from their social networks (i.e., friends, family, peers). One female survivor commented on her partner’s deliberate use of COVID-19 movement restrictions to cut her off from her social network:

*I think he tried to push people away, you know, like* [friend’s name] *said... My best friend, she came over to the house, and she was like, “I could stand on one side of the fence, man, you could stand on the other side of the fence in your yard, and we could chop it up. I’ll eat on my car, and you could eat on your car.“ And he wasn’t going for it, even though we were six feet apart. So, I don’t think it was COVID related. I strongly feel that it was an opportunity to, like I said, tighten those reigns. (39-year-old Multiracial female survivor)*

Across interviews survivors indicated this isolation resulted in larger amounts of time spent with their partners, prompting more opportunities for abuse.

#### COVID-19 restrictions worsened IPV through increased financial and life stressors

The pandemic also affected survivors’ experiences of IPV relative to financial and life stressors stemming from COVID-19 movement restrictions and their impacts. Examples of such stressors include, economic, housing, and job instability, stress resulting from remote employment, virtual schooling for children, potential COVID-19 exposure, and relationship strain created by conflicts over the use of government stimulus payments. Across all cases, survivors discussed intensified and high frequencies of arguments in their relationships during the pandemic. Among the six relationships that existed prior to the pandemic all survivors indicated the frequency and intensity of arguments increased; this high frequency of arguments and subsequent relationship strain contributed to IPV episodes. One survivor noted that following the onset of the pandemic, her relationship became more violent:

*“We argue more after COVID-19 than we did before COVID-19. But to be honest, if I really had to compare the two, we argued the same, things just got worse, like he started really, putting his hands on me after that.” (23-year-old Black/African American female survivor)*

Several survivors cited financial stress or economic instability during the pandemic as a key feature of their relationship challenges. For example, one survivor noted both her and her partner lost their jobs during the pandemic and had to move in with her mother due to loss of housing:

*“I noticed that everything started to decline, he started getting stressed about things. You know, he wasn’t happy, and um, money was the issue. You, know, again, I was back in my mom’s house, he was there, too. So, you know, we just wanted to try to find [a job], that was worth it, but he was so stuck on being a cook, you know, that I was stuck on getting something bigger and better and he wasn’t.” (31-year-old Black/African American female survivor)*

In this example, financial challenges during COVID gave way to more arguments and episodes of IPV because of the perpetrator’s high spending on alcohol.

Additionally, job or housing instability resulting from the impacts of COVID-19 contributed to relationship challenges. A number of survivors described how they and/or their partners lost employment or were furloughed during the pandemic, were evicted, or had to move in with their families. Such instability, in turn, contributed to the financial strain within relationships.

COVID-19 movement restrictions and social distancing measures resulting in remote work status and/or virtual schooling for children also fueled arguments and relationship challenges. Some survivors indicated they had to work remotely while their children also began virtual schooling and their partners worked outside the home as essential workers. Subsequently, working from home coupled with increased caregiving responsibilities or disputes over child discipline, resulted in added relationship strain. One survivor explained,

*“Um, but COVID…I think made it more, um, of a bigger issue for me, especially ‘cause I’m like, okay, you’re coming in here asking about cleaning or whatever. Meanwhile, I’m managing kids that are acting crazy and my job...You know, I already told you that it was stressful. So, I’m like, you already know my work situation. So, I don’t have time to like... It, it’s a lot trying to keep the kids together and do my work. So, why are you...being a jerk about, why isn’t the house clean ...So, I think for me, that um, became another layer of resentment.” (45-year-old Black/African American female survivor)*

Survivors noted novel or increased episodes of IPV during the pandemic as a result of this relationship strain. Several survivors noted increased disagreements with partners regarding breaking movement restrictions or potential COVID-19 exposure. Regarding perpetrators who worked outside the home as essential workers during the pandemic, survivors noted increased arguments and relationship strain attributed to fear and anxiety concerning their partners’ potential exposures to COVID-19 while working. For example:

*“So, she had to travel for work. She has the, what do they call them…Yeah, the essential worker…And so she had to go out of town a lot. She had to go to work and come back home. And. And I was scared because, you know, high risk is on my children.” (36-year-old Black/African American female survivor)*

Other types of relationship strain or violence stemming from breaking movement restrictions pertained to one partner, usually the survivor, disagreeing with the other partner’s lack of adherence to COVID-19 precautions. Among these participants, partners in the relationship differed in their COVID-19 risk tolerance resulting in disagreements about how to operationalize COVID-19 mitigation strategies such as social isolation. In several cases, disagreements over leaving the house during the pandemic resulted in episodes of physical IPV. One survivor indicated:

*“It got worse after COVID because I tried to keep her isolated [socially distanced], out the street, but…She couldn’t stay away from that old neighborhood…A lot of people was dying in that area from COVID. You know, and I don’t want her to running over there and running back into my [COVID-19] isolation zone.“ (65-year-old Black/African American male survivor)*

Furthermore, some survivors noted increased relationship challenges or strain resulting from government stimulus payments, also known as stimulus checks. These relationship challenges pertained to disagreements concerning how to use the money. For example, one survivor noted her partner feared she would take his stimulus money and use it for herself and end the relationship while he spent the money: *“It seems like every time a check would roll around, there was, there was a breakup” (37-year-old White female survivor).*

Collectively, novel or preexisting relationship challenges amplified by the impacts of the pandemic contributed to episodes of IPV due to more frequent arguments. Although some survivors indicated they did not believe the pandemic directly contributed to their relationship issues or IPV, they still described challenges that affected their relationships negatively.

### COVID-19 movement restrictions catalyzed new relationships quickly and sparked new or intensified violence in existing relationships

COVID-19 movement restrictions appeared to impact the trajectory of both new and existing relationships. Dimensions of this theme include new instances of IPV occurring in relationships that started during the pandemic, new instances of IPV in existing relationships, and intensified instances of IPV in existing relationships. Movement restrictions, social distancing measures, and the negative repercussions of the pandemic influenced the amount of time couples spent together, their relationship challenges, as well as the occurrences of new, more frequent and/or severe IPV episodes.

#### COVID-19 movement restrictions catalyzed relationships and triggered new violence

Four survivors discussed their pandemic partnerships — relationships that began and ended during the pandemic. Notably, two survivors indicated their relationships began during March 2020, when movement restrictions and social distancing measures were enacted, and ended in July of 2020 when restrictions were relaxed in Georgia. Common descriptions of this type of relationship included spending large amounts of time with each other daily, moving in together after dating for a few weeks due to movement restrictions, fear of COVID-19 exposure, financial strain, or job instability. When asked to describe how they thought the pandemic influenced their relationship and overall perception of relationship safety, one survivor underscored the importance of access to their social network:

*“Like the exposure to other people, and I think it’s easy to like, have that gaslighting in their relationship and kind of feel like everything’s normal, um, if you’re not exposed to other people or having that connection with other people…I think that if COVID hadn’t happened [then] my relationship would have looked very differently…it’s progressed my relationship into a serious relationship very quickly.” (22-year-old Multiracial female survivor)*

One survivor in a pandemic partnership noted how they spent increased time together: *“I did see them like gradually…we’ll spend like a whole day or two together, like, like as much as 48 hours or 24 hours” (22-year-old Black/African American non-binary survivor).*

Two survivors in pandemic partnerships discussed moving in with their partners after briefly dating due to fear of COVID-19 exposure or instability created by the pandemic. One survivor was particularly fearful of exposure to COVID-19, stating:

*“But during the pandemic, um, I started seeing stuff, and he would leave out the house, you know, and he asked me cause we was in a situation, he asked me, well let me just come stay with you, cause you tell me, you know, every time I come over, I gotta be tested, so if I stay here wit you, you know, you’ll feel better. And I’m like yeah, get tested [inaudible], I did that, I let him stay...Well, after we started datin’, um, I moved him in because I was like, you know, I like you, you get along with my dog.” (54-year-old Black/African American female survivor)*

Survivors also indicated that as their short relationships progressed arguments increased. Survivors in pandemic partnerships also discussed experiencing IPV, either physical or psychological, for the first time ever. When asked if she had experienced fights or arguments with her partner or previous partners prior to an episode of physical IPV where her partner punched her in the face, one survivor noted this was the first time she experienced physical violence in any relationship, resulting in her desire to terminate the relationship. She went on to discuss a subsequent episode of physical IPV occurring only a few days later, in which the perpetrator cut her arm with a knife, requiring paramedic intervention:

“*It was a couple of days afterwards, actually, when we got into a tussle, um, we tried to come back, recon-reconcile together, but that didn’t work and it ended very badly. So, um, we were in a tussle and, um, for my protection, I grabbed a knife, and it was just swinging everywhere and, um, he saw me grab the knife and he pressed the knife against my arm and it scraped against my skin. And, um, I was just bleeding very, very badly.” (31-year-old Black/African American Female survivor)*

#### COVID-19 movement restrictions sparked new or intensified violence in existing relationships

Survivors in relationships that existed prior to the pandemic frequently discussed new or exacerbated relationship challenges, arguments, or IPV following the onset of the pandemic. Regarding her long-term relationship, one survivor noted:

*“It was abusive. COVID didn’t make it any, um... I mean, CO-...COVID didn’t produce the abuse, the abuse was already pre-existing. It just got worse during COVID.” (39-year-old Multiracial female survivor)*

Other survivors noted the frequency of arguments with their partners increased from a couple per week prior to COVID, to every single day during the pandemic. Following increased relationship strain, several survivors indicated experiencing episodes of IPV for the first time, physical IPV requiring hospitalization for the first time, or more intense IPV episodes following the onset of COVID-19. When asked if this was the first time she experienced relationship violence requiring hospitalization, a survivor indicated: *“Yes, in my life, never once experienced something like this in my life” (23-year-old Black/African American female survivor).*

Those in pandemic partnerships described accelerated relationship timelines stemming from increased free time and fear of COVID-19 exposure resulting from movement restrictions and pandemic repercussions. Conversely, survivors in existing relationships described new or exacerbated relationship challenges or arguments stemming from the pandemic-related movement restrictions and life impacts. Collectively, the impacts of COVID-19 laid the foundation for new or intensified violence within both pandemic partnerships and existing relationships.

## Discussion

The purpose of this study was to understand the impacts of COVID-19 and related movement restrictions (i.e., shelter-in-place orders, quarantine, isolation orders), on IPV from the perspective of survivors. Survivors discussed a variety of trauma experiences resulting from IPV and how COVID-19 movement restrictions impacted their experiences and help-seeking behaviors. All survivors discussed relationship challenges that were amplified by either movement restrictions or consequences of COVID-19, supporting other research on IPV that found increases during the pandemic [41].

Survivors drew connections between COVID-19 movement restrictions and their partner’s control or abuse tactics. For these survivors, movement restrictions (i.e., shelter-in-place orders, school closures, curfews) trapped them with their abusers by creating isolating environments, which they felt exacerbated coercive control and other abuse tactics [4–6]. Survivors also indicated increased financial or life stressors resulting from COVID which featured prominently in their IPV experiences. Notably, survivors attributed these COVID-19-related relationship challenges to higher recurrences of arguments or fights, which often preceded episodes of IPV, reinforcing preliminary evidence pointing to increases in IPV and DV, especially during lockdown and social distancing periods [1–3]. These data are consistent with research on IPV during natural disasters and other health emergencies [42–45].

Survivors also mentioned relationship challenges and subsequent IPV experiences driven by unbalanced caregiving burdens for children. Specifically, a few female survivors had to balance virtual work requirements and the supervision of their children’s virtual schooling whilst their abuser continued to work outside the home throughout the pandemic. These findings bolster prior research establishing unequal caregiving burdens placed on women during the pandemic [46, 47]. Other research connecting lockdown and economic stress factors to increases in IPV indicates lifted movement restrictions will not decrease IPV and DV due to continued economic stress fueled by the pandemic, suggesting a new normal for IPV perpetration [2, 3, 8, 48, 49].

COVID-19 created a new reality discussed by survivors as stemming from unemployment, remote work environments, movement restrictions, and social distancing measures, allowing intimate partners to spend more time with each other than they might have pre-pandemic. The unique situation and environment created by the pandemic were described by survivors as impacting the trajectory of their relationships, both new and existing. Through discussion of their experiences, survivors indicated movement restrictions, social distancing measures, and the negative repercussions of the pandemic influenced their relationship challenges, as well as the occurrences of new or a higher frequency and/or severity of IPV episodes. Their experiences further bolster prior research suggesting the pandemic increases the likelihood of experiencing IPV and DV, especially during shelter in place orders [1–3, 8, 48, 49].

Taken as a whole, these findings suggest COVID-19 movement restrictions and social distancing measures amplify IPV and experiences of trauma. New and worsened experiences of IPV documented by this study, COVID-19 research, as well as IPV research conducted during previous health emergencies (i.e., Ebola Virus Disease epidemic) suggest that the interaction between IPV and movement restrictions is not unique to COVID-19 [42–45]. Thus, this phenomenon and its impacts must receive serious consideration when enacting movement restrictions during future pandemic response.

As the pandemic continues to threaten public health and safety, findings from this study can be leveraged to inform IPV response during the ongoing pandemic as well as future public health emergencies. As indicated by survivors, increased visibility and overall availability of more IPV resources beyond emergency housing, access to free or subsidized counseling or support groups, as well as increased use of virtual resource provision would bridge gaps and obstacles created by the pandemic and movement restrictions. Specifically, several survivors interviewed for this study referenced financial and housing instability as drivers of conflict and subsequent IPV experiences. Safe housing and economic resources have been documented as the top concerns for survivors who are planning to leave or have already left abusive partners [50, 51]. Although federal relief efforts were enacted to offset negative repercussions of the pandemic, these efforts did not arrive until later in the pandemic. For example, the Emergency Rental Assistance program was not established until December 2020, nine months after the pandemic began in the US [52]. As several survivors cited moving in with newer partners due to financial or housing instability and experiencing IPV during the early months of the pandemic, the importance of the availability and timely provision of financial/housing support for IPV survivors during public health emergencies is further supported by study; the need for safe housing was likely worsened by limited shelter housing, an existing problem, coupled with the need to socially distance shelter residents limiting already strained shelter capacity.

As the survivors in our study came from both hospital and community settings, we were able to hear about the effects of the pandemic on their relationship experiences relative to where they sought care. Future research may explore the opportunities for connecting hospital and community-based care to further ensure survivor safety. Moreover, findings can also be used to inform future pandemic preparedness and response among IPV and public health resources in Atlanta and the state of Georgia. The methods carried out in this study can be adapted for future research carried out in other areas of Georgia or the broader US pertaining to IPV experiences during COVID-19 and perceptions of IPV survivors. Future research should also examine the effects of federal financial and housing assistance efforts on IPV survivors’ experiences, in addition to survivor help-seeking behaviors and experiences as movement restrictions (i.e., stay-at-home orders, social distancing measures) were lifted during the protracted phase of the pandemic.

There are several limitations to note for this study. Initially, the study aimed to recruit 30 survivors by applying a novel natural language processing algorithm to identify survivors from among patient records from a large public hospital in Atlanta, Georgia. While we were able to identify a large sample of participants, we faced significant challenges in recruitment. As a result, we expanded recruitment to include IPV survivors from across Metropolitan Atlanta, resulting in a final sample of ten survivors derived from hospital- and community-based recruitment. Recruitment challenges may be attributed to the hidden nature of IPV survivors and the sensitive topic of the study. Likewise, our eligibility criteria required that IPV care was sought between March-December 2020 making recruitment more challenging as time progressed and as the pandemic continued. Due to the nature of qualitative research, results cannot be generalized to the entire population of IPV survivors. Additionally, a majority of survivors identified as cisgender, heterosexual, and African American/Black. Therefore, we are largely missing perspectives from other racial, gender, and sexual identities. However, our sample of mostly African American/Black survivors adds value given the disproportionate burden of COVID-19 on Black, Indigenous and other people of color and the fact that our data were collected during a period of racial reckoning in the U.S. [41, 53–55]. Yet, because our original research question did not center around racial differences and IPV experiences during the pandemic we were unable to explore the effect of individualized or structural racism on survivors’ experiences related to COVID-19 or IPV. Although the codebook was created collaboratively with the entire research team, there was only one coder for data analysis. Additionally, there is potential sample bias, as we only interviewed survivors who sought IPV services during the early months of the pandemic. Therefore, we are missing perspectives of IPV survivors who sought support services or resources beyond this time period or those who did not seek services at all. As such, findings from this study should be complemented by expanding data collection to incorporate more voices from IPV survivors in Georgia and other regions of the US, as well as those who sought care during other time periods of the pandemic.

## Conclusion

Since the start of the pandemic, anecdotal and subsequently cross-sectional empirical evidence have documented surges in IPV and IPV help-seeking among survivors worldwide [10–16]. In research examining COVID-19 and IPV that has emerged in the past two years, several systematic reviews point to increases in IPV and domestic violence, especially during lockdown and social distancing periods [1–3]. As movement restrictions and social distancing practices fade from practice —and the pandemic continues—the long-term effects of the pandemic on IPV, and in particular survivor experiences, are still largely unknown. This study addresses the gap in knowledge about IPV survivors’ perceptions of COVID-19 movement restrictions (i.e., shelter-in-place, quarantine, isolation orders) and their effects on experiences of relationship violence. Findings from this study contextualize survivors’ experiences of IPV during the COVID-19 pandemic, facilitators of IPV experiences during COVID-19, as well as changes in experiences of IPV from before and during the pandemic. Documenting and comprehending survivors’ experiences and perceptions, particularly situations in which infection isolation policies mimic or reinforce coercive control by abusive partners or catalyze relationship violence, offers a means to explore the connection between COVID-19 and IPV. Such an understanding may result in improved IPV prevention and response tactics implemented during this pandemic, as well as the long-term changes to IPV experiences that may continue in a post-pandemic world.

## Data Availability

Due to privacy, safety, and ethical concerns, supporting data cannot be made openly available; deidentified data may be made available upon reasonable request. Data requests should be made to Dr. Dabney P. Evans at devan01@emory.edu.

## Authors’ information

Emory University Rollins School of Public Health, 1518 Clifton Rd, Atlanta 30322, USA.

## Abbreviations

IPV: Intimate partner violence
US: United States
UK: United Kingdom
IDI: In-depth interview
ICD: International Classifications of Disease
